# Intimate partner violence and covert contraception use among Ugandan women: a cross-sectional inquiry

**DOI:** 10.1186/s12978-026-02425-7

**Published:** 2026-08-12

**Authors:** Craig J. Heck, Kyle Wu, Hadijja Nakawooya, Tom Lutalo, Seda Shirinian, Fred Nalugoda, John Santelli, Jennifer Wagman

**Affiliations:** 1 Division of Infectious Diseases, Department of Medicine, Columbia University Irving Medical Center, 622 West 168th Street, New York, NY 10032 USA; 2 Department of Community Health Sciences, UCLA Fielding School of Public Health, 650 Charles E. Young Dr. South Room 46-071B CHS, Los Angeles, CA 90095 USA; 3 Rakai Health Sciences Program, Old Bukoba Road, Kalisizo, Uganda; 4 Heilbrunn Department of Population and Family Health, Columbia University Mailman School of Public Health, 722 West 168th St, New York, New York 10032 USA

**Keywords:** Gender inequity, Family planning, Reproductive rights, Reproductive coercion, Secret contraceptive use

## Abstract

**Introduction:**

Intimate partner violence (IPV) is prevalent globally, adversely impacting women’s health and autonomy. Covert contraceptive use (CCU), using contraception without a partner’s knowledge, may arise in response to IPV and restrictive gender norms. This study examines associations between verbal, physical, and sexual IPV and CCU among Ugandan women, providing insight into the socio-cultural dynamics influencing reproductive health choices.

**Methods:**

We conducted a secondary analysis using data from the 2011–2013 Rakai Community Cohort Survey, which collected demographic, reproductive, and IPV-related data from women in South-Central Uganda. We included women who reported (1) ever having sex, (2) no current pregnancies, and (3) current use of contraception, including intra-uterine devices, pills, injectables, or implants. Adjusted prevalence ratios (APR) and 95% Confidence Intervals (CI) were estimated using Poisson regression, adjusting for age, education, marital status, HIV status, alcohol use, and other confounders.

**Results:**

Among 2,094 women, 27%, 21%, and 12% respectively survived verbal, physical, and sexual IPV, and 26% practiced CCU. Positive IPV-CCU associations arose for physical IPV (APR = 1.39, CI = 1.17–1.65) and verbal IPV (APR = 1.30, CI = 1.12–1.52), whereas sexual IPV’s association was marginally significant (APR = 1.22, CI = 0.99–1.49).

**Conclusions:**

IPV may influence women’s choices about contraception and/or women’s use of contraception may precipitate IPV. Integrating efforts to prevent IPV and provide contraception are needed to better serve women.

**Supplementary Information:**

The online version contains supplementary material available at https://doi.org/10.1186/s12978-026-02425-7.

## Introduction

In 2015, the World Health Organization introduced the Agenda for Sustainable Development, which outlined 17 goals aimed at improving health, preserving environments, increasing prosperity, promoting peace, and mobilizing partnerships by 2030 [1]. Sustainable Development Goal 5’s commitment to achieving gender equality includes targets on eradicating reproductive coercion and gender-based violence. However, current evidence suggests that progress toward these goals is lagging: 53% of married or partnered women in Eastern Africa independently make sexual and reproductive health (SRH) decisions and one-in-four women experience physical and/or sexual intimate partner violence (IPV) in their lifetime [2, 3].

Amid imbalanced relationship power, cisgender women (hereafter referred to as women) may practice covert contraceptive use (CCU), use without partners’ knowledge, to regain autonomy of SRH decisions [4–6]. According to Demographic and Health Survey estimates, CCU in East Africa ranges from 5.4% in Zambia (2018) to 12.8% in Uganda (2016) [7]. Uganda stands out in particular because of its relatively high and persistent prevalence of CCU compared to other East African countries, with nationally representative data from 1995 to 1996 and 2019–2020 reporting a CCU prevalence of 14.8% and 13.1%, respectively [8, 9]. This high rate, coupled with socio-cultural factors such as patriarchal norms and limited reproductive autonomy for women, makes Uganda a particularly relevant setting for investigating the dynamics between IPV and CCU [10–12]. National estimates, however, may obscure regional differences, as one-in-four women reported CCU in an open community cohort in Rakai District, Uganda [13]. Understanding the persistence and drivers of CCU in Uganda could offer key insights for addressing gender inequalities in reproductive health across the region.

Gender-inequitable norms implicitly link IPV and CCU. Men view CCU negatively because it can interfere with their fertility desires; thus, discovering CCU may lead them to perpetrate verbal, physical, and/or sexual IPV [6, 14]. Conversely, IPV may compel women to practice CCU to prevent unplanned or unwanted pregnancies, whereas in other cases, IPV may deter contraceptive use entirely [14, 15]. Gender norms also complicate women’s CCU views and practices. For example, qualitative evidence from India reveals that some women perceive marital IPV as acceptable when wives eschewed their expectations, including preventing childbearing via CCU [16]. Given the complexities of these dynamics, the relationship between IPV and CCU warrants further investigation. The breadth and depth of the IPV-CCU intersection remain poorly understood due to a dearth of quantitative research, particularly in Uganda, the East African country with the highest CCU prevalence [7]. In a Nigerien study, physical IPV was positively associated with CCU among married adolescent girls, but no such association was found with sexual IPV [17]. Our previous research in Uganda identified verbal, physical, and sexual IPV as positive unadjusted correlates of CCU, with physical IPV emerging as the only adjusted association [13]. A more detailed analysis of the different types of IPV may better clarify their individual impacts on CCU, as various other factors—including age, education, religion, occupation, relationship dynamics, number of sex partners, HIV status, and alcohol use—may confound these associations [16, 18–22]. Therefore, this study aimed to individually estimate the adjusted association of verbal, physical, and sexual IPV on CCU among women enrolled in an open population-based community cohort in southwestern rural Uganda.

This study is a secondary analysis of data collected in South-Central Uganda from 2011 to 2013 through the Rakai Community Cohort Survey, one of the oldest longitudinal population-based HIV and reproductive health cohort studies in the world. Given that IPV is a common marker of gender and relationship power differentials, we hypothesized that CCU would be more prevalent among those who have experienced or survived each type of IPV.

## Methods

### Study design

This secondary analysis draws data from the Rakai Community Cohort Survey (RCCS), whose methods are detailed elsewhere [23, 24]. Briefly, RCCS is designed to monitor health outcomes, particularly related to the HIV epidemic and sexual and reproductive health, in 30 + communities throughout the Rakai region of South-Central Uganda. RCCS is an open, prospective population-based cohort that enrolls consenting adult residents aged 15–49 years. The specific data used in this analysis come from RCCS Round 15 (R15), collected between 2011 and 2013. At each survey round, trained same-sex study staff privately administered structured surveys in Luganda, the local language. These surveys collected self-reported data on demographics, fertility, reproductive health outcomes, contraceptive use and sexual behaviors. New RCCS enrollees completed a baseline survey, while continuing RCCS participants completed the follow-up survey. Study staff also collected blood specimens for HIV testing, which was used as a covariate in this analysis.

This article adheres to the reporting standards established by the Strengthening the Reporting of Observational Studies in Epidemiology (STROBE) Statement (Supplemental Material 1) [25].

### Sample

The sample was restricted to RCCS’ R15 because it was the first to ask about CCU. Additional inclusion criteria comprised (1) female sex; (2) using contraceptive injectables, intrauterine devices (IUDs), implants, or pills; (3) non-missing CCU data; (4) not pregnant; and (5) having ever had sexual intercourse.

### Dependent variable

All females using contraceptive injectables, IUDs, implants, or pills were asked: “Does your partner know that you are using this method of family planning?”, with “No” responses reflecting a positive CCU indication; “Yes” responses comprised the reference group. This operational definition of CCU as contraceptive use without the partner’s knowledge is aligned with existing research on covert contraceptive use.

### Independent variables

#### Sociodemographics

Age was categorized into four groups: 15–24, 25–29, 30–34, 35–49. Educational attainment was categorized as None, Primary, Post-primary. Religion was grouped as Catholic, Protestant, Pentecostal, Muslim or Other. Occupation was categorized as agriculture, housework, professional service or other. Marital status was defined as never married, married or previously married. These variables were chosen because they are well-established correlates of both IPV and contraceptive use.

#### Biological

HIV status was measured using two ELISA and confirmatory HIV-1 Western blot tests. Results were qualitatively captured as Positive or Negative [26]. HIV status was included as a covariate given the strong associations between HIV risk and reproductive decision-making behaviors.

#### Behavioral

Contraceptive methods assessed were those included in the survey’s skip pattern for the CCU question: contraceptive injectables, IUDs, implants, or pills. Participants were asked about the number of sexual partners they had in the last 12 months, which was dichotomized as 1 vs. >1. Alcohol use was measured with binary variables capturing respondents’ and partners’ use of alcohol before sex. Alcohol use is relevant to this analysis as it is known to affect both sexual decision-making and IPV.

#### IPV

All IPV questions were framed with a recall period of the last 12 months and were adapted from the Conflict Tactics Scales (CTS) [27]. Verbal IPV was measured by asking if a husband/partner verbally abused or shouted at the respondent, in line with the CTS definition of psychological aggression. Physical IPV was captured using an ordinal variable adapted from the CTS: Severe (husband/partner attempted to strangle or burn the respondent or threatened or attacked them with knife, gun, or other weapon); Moderate (husband/partner pushed, slapped, held down, punched, kicked, or dragged respondent); or None. Sexual IPV, also adapted from the CTS, was measured by asking if a sexual partner had used verbal threats, physical force, or other means to force the respondent to have or perform unwanted sex.

These specific IPV measures were selected because they encompass the key dimensions of IPV (verbal, physical, and sexual) and reflect the experiences most likely to influence reproductive decision-making.

### Statistical analysis

Analyses were conducted in R (v.4.3.1) [28, 29]. We reported descriptive statistics using proportions. Unadjusted and adjusted prevalence ratios and 95% Confidence Intervals (CI) were estimated using Poisson regression models with sandwich variance estimators. Poisson regression was chosen as the appropriate model to estimate prevalence ratios for the binary outcome (CCU). Along with IPV measures, all adjusted models controlled for age, education, religion, occupation, marital status, HIV status, contraceptive method, multiple partnerships, and alcohol use. Ordinal independent variables were analyzed using orthogonal polynomial coding to assess the linear trend across ordered categories. Statistical significance was assessed using α = 0.05. We also examined multicollinearity between IPV types by computing polychoric correlation and calculating generalized variance inflation values for each independent variable using sandwich variance estimators.

## Results

Of 9,233 females, 3,265 used contraceptives; of which, 67% (2,199/3,265) used contraceptive injectables, IUDs, implants, or pills. After removing 88 respondents for not responding to the CCU question and 17 for never having sex, the final analytic sample included 2,094 females.

### Sample characteristics

Table 1 provides a detailed overview of the sample characteristics. Age distribution was relatively even across the 15–24, 25–29, 30–34, and 35–49 age groups. Most respondents had attained a primary-level education (61%), worked in agriculture (40%) or the professional service industry (32%), identified as Catholic (65%), and reported being married (76%). 22% were living with HIV, 76% used injectable contraceptives, and 90% had only one partner in the last 12 months. Partners’ alcohol before sex (44%) was reported twice as frequently as respondents’ own alcohol use (21%).

**Table 1:** Sample characteristics (*N* = 2094)

	Overall % (*n*)
Age groups	
15–24	25% (524)
25–29	28% (575)
30–34	25% (531)
35–49	22% (464)
Highest education level	
None	4% (84)
Primary	61% (1,281)
Post Primary	35% (728)
Religion	
Other	1% (16)
Catholic	65% (1,350)
Protestant	16% (341)
Pentecostal	3% (68)
Muslim	15% (318)
Occupation	
Agriculture	40% (836)
Housework	9% (197)
Other	7% (151)
Professional	12% (248)
Service	32% (662)
Marital status	
Never married	7% (150)
Married	76% (1,593)
Previously married	17% (351)
HIV status	
* Negative*	78% (1,634)
* Positive*	22% (451)
Contraceptive method used	
Injectables	76% (1,583)
IUD/Coil	5% (105)
Implants	7% (139)
Pills	13% (267)
Number of sexual partners in last 12 months	
One	90% (1,876)
More than one	10% (215)
Partner Drank Before Sex	44% (911)
Respondent Drank Before Sex	21% (440)

Missing data for each variable was < 1%

### IPV prevalence

**Fig. 1:**
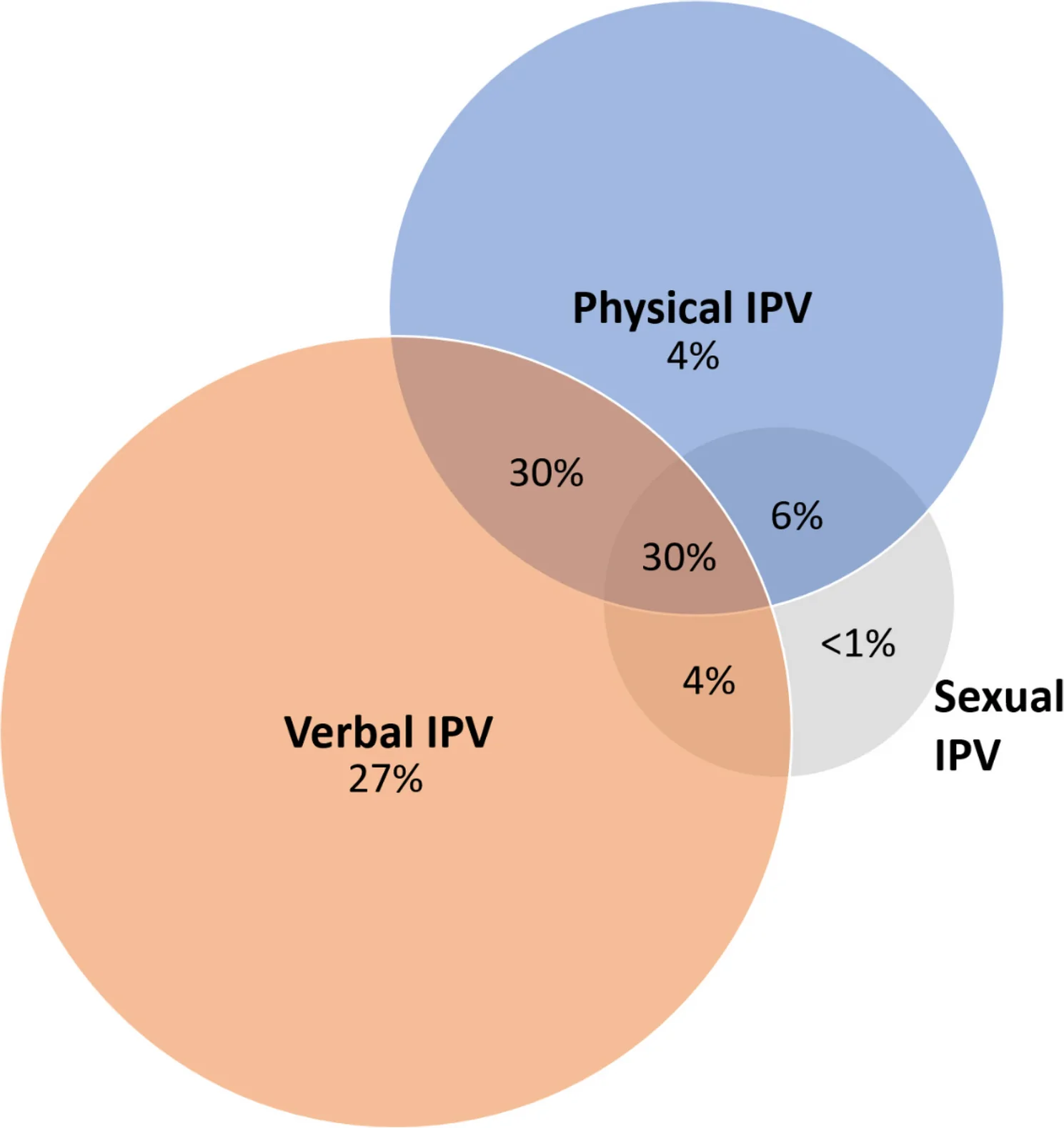
Intersections of verbal, physical, and sexual IPV among survivors (*N* = 621) Total percentage exceeds 100% due to rounding. Circles are sized to reflect the prevalence of each IPV type among survivors (Verbal: 90%, Physical: 70%, Sexual: 39%)

Overall (*N* = 2,094), 30% (*n* = 621) experienced at least one form of IPV. By type, 27% (*n* = 561) reported verbal IPV, 21% (*n* = 432) reported physical IPV (17% [*n* = 353] moderate, 4% [*n* = 79] severe), and 12% (*n* = 245) reported sexual IPV. Polyvictimization, surviving multiple forms of IPV, was also frequently reported. Figure 1 illustrates the overlap between different IPV types among survivors with complete violence data (*N* = 621). Of which, 30% (*n* = 188) reported verbal and physical IPV, 30% (*n* = 185) reported all three IPV types, 27% (*n* = 165) reported verbal IPV alone, and 6% (*n* = 36) reported physical and sexual IPV. Other IPV combinations were rare (< 5%).

### Associations between verbal, physical, and sexual IPV survivorship and CCU

One-fourth of the sample (26%) practiced CCU. Table 2 presents the unadjusted associations between verbal, physical, and sexual IPV survivorship and CCU, with all three IPV types exhibiting a positive association.

**Table 2:** Cross-tabulations and unadjusted associations of IPV types and other factors with CCU

	Covert contraceptive use		
	No (*n* = 1556)	Yes (*n* = 538)	PR (95% CI)
**Independent variables of interest**			
Experienced verbal IPV	24%	34%	**1.41 (1.21–1.64)**
Severity of physical IPV			**1.51 (1.24–1.84)**
None	82%	72%	
Moderate	15%	22%	
Severe	3%	6%	
Experienced sexual IPV	11%	15%	**1.36 (1.12–1.65)**
**Control variables**			
Age groups			
15–24	25%	25%	1.00 (ref)
25–29	27%	29%	1.04 (0.85–1.27)
30–34	26%	25%	0.97 (0.79–1.19)
35–49	23%	21%	0.95 (0.76–1.17)
Highest education level			0.64 (0.52–0.79)
None	3%	7%	
Primary	61%	63%	
Post Primary	36%	30%	
Religion			
Other	1%	1%	1.00 (ref)
Catholic	65%	64%	1.35 (0.49–3.76)
Protestant	16%	16%	1.38 (0.49–3.88)
Pentecostal	3%	4%	1.65 (0.56–4.85)
Muslim	15%	16%	1.41 (0.50–3.97)
Occupation			
Agriculture	43%	32%	1.00 (ref)
Housework	9%	10%	1.36 (1.05–1.76)
Other	6%	10%	1.64 (1.27–2.13)
Professional	12%	11%	1.14 (0.88–1.48)
Service	30%	38%	1.48 (1.24–1.77)
Marital status			
Never married	6%	10%	1.00 (ref)
Married	81%	62%	0.58 (0.46–0.73)
Previously married	13%	28%	1.21 (0.94–1.54)
HIV status			
Negative	81%	72%	1.00 (ref)
Positive	19%	28%	1.43 (1.23–1.68)
Contraceptive method used			
Injectables	76%	75%	1.00 (ref)
IUD/Coil	4%	7%	1.34 (1.02–1.77)
Implants	7%	6%	0.90 (0.66–1.24)
Pills	13%	12%	0.97 (0.77–1.21)
Number of sexual partners in last 12 months			
One	92%	83%	1.00 (ref)
More than one	8%	17%	1.80 (1.52–2.15)
Respondent Drank Before Sex	19%	26%	1.31 (1.11–1.54)
Partner Drank Before Sex	42%	49%	1.26 (1.09–1.46)

*PR* Prevalence Ratio

PRs for physical IPV severity and highest education level reflect linear polynomial estimates

Column percentages are presented; percentages may slightly exceed 100% due to rounding

For the independent variables of interest, bolded measures indicate *p* ≤ 0.05

When all three IPV variables were included in the same model, multicollinearity assessment revealed polychoric correlation coefficients ≥ 0.79 for all pairwise combinations, indicating strong positive correlations (Supplemental Material 2). Due to this high multicollinearity, individual adjusted models are presented for verbal, physical, and sexual IPV. 

Table 3 contains the adjusted associations for verbal, physical, and sexual IPV survivorship: after adjustment, the verbal and physical IPV associations remained statistically significant (*p* ≤ 0.05), while sexual IPV’s relationship trended toward significance (*p* = 0.0505). Of the three adjusted models, physical IPV had the strongest measure of association, as survivors were 1.39-times as likely (95% CI: 1.17–1.65) to practice CCU than those who did not report physical IPV. Additionally, those who experienced verbal IPV were 1.30-times as likely (95% CI: 1.12–1.52) to report CCU. Sexual IPV survivors were 1.22-times as likely to practice CCU (95% CI: 0.99–1.49), but this relationship was only marginally significant. None of the individual models had generalized variance inflation values that indicated multicollinearity concerns (data not shown).

**Table 3:** Adjusted associations between IPV types and CCU

	Practiced covert contraceptive use		
	APR (95% CI)	APR (95% CI)	APR (95% CI)
**Independent variables of interest**			
Verbal IPV	**1.30 (1.12–1.52)**		
Increasing severity of physical IPV		**1.39 (1.17–1.65)**	
Sexual IPV			1.22 (0.99–1.49)
**Control variables**			
Age groups			
15–24	1.00 (ref)	1.00 (ref)	1.00 (ref)
25–29	1.04 (0.85–1.26)	1.02 (0.83–1.24)	1.02 (0.83–1.24)
30–34	0.95 (0.77–1.18)	0.94 (0.76–1.16)	0.93 (0.75–1.15)
35–49	0.94 (0.74–1.18)	0.93 (0.74–1.17)	0.91 (0.72–1.14)
Increasing education level	0.74 (0.60–0.91)	0.74 (0.60–0.92)	0.73 (0.60–0.90)
Religion			
Other	1.00 (ref)	1.00 (ref)	1.00 (ref)
Catholic	1.28 (0.56–2.96)	1.32 (0.56–3.08)	1.26 (0.55–2.94)
Protestant	1.30 (0.56–3.03)	1.32 (0.56–3.13)	1.27 (0.54–2.98)
Pentecostal	1.56 (0.63–3.87)	1.60 (0.64-4.00)	1.55 (0.62–3.85)
Muslim	1.44 (0.61–3.37)	1.47 (0.62–3.50)	1.42 (0.60–3.35)
Occupation			
Agriculture	1.00 (ref)	1.00 (ref)	1.00 (ref)
Housework	1.28 (0.98–1.67)	1.28 (0.98–1.68)	1.31 (1.00-1.71)
Other	1.35 (1.04–1.75)	1.36 (1.05–1.77)	1.36 (1.05–1.77)
Professional	1.16 (0.87–1.54)	1.16 (0.88–1.54)	1.17 (0.88–1.56)
Service	1.20 (0.99–1.45)	1.20 (0.99–1.45)	1.22 (1.01–1.47)
Marital status			
Never married	1.00 (ref)	1.00 (ref)	1.00 (ref)
Married	0.54 (0.42–0.68)	0.55 (0.43–0.70)	0.56 (0.44–0.71)
Previously married	0.98 (0.75–1.27)	0.99 (0.77–1.29)	1.01 (0.78–1.31)
HIV status			
Negative	1.00 (ref)	1.00 (ref)	1.00 (ref)
Positive	1.10 (0.93–1.30)	1.10 (0.93–1.30)	1.13 (0.95–1.33)
Contraceptive method used			
Injectables	1.00 (ref)	1.00 (ref)	1.00 (ref)
IUD/Coil	1.45 (1.08–1.94)	1.46 (1.09–1.96)	1.47 (1.10–1.97)
Implants	0.98 (0.71–1.35)	0.95 (0.69–1.30)	0.99 (0.72–1.36)
Pills	0.93 (0.75–1.16)	0.93 (0.75–1.15)	0.93 (0.74–1.15)
Multiple sex partners in last 12 months	1.31 (1.09–1.58)	1.31 (1.08–1.58)	1.33 (1.10–1.61)
Respondent Drank Before Sex	0.99 (0.82–1.19)	0.97 (0.80–1.16)	1.00 (0.83–1.20)
Partner Drank Before Sex	1.23 (1.05–1.44)	1.23 (1.05–1.45)	1.25 (1.06–1.47)

*APR* Adjusted Prevalence Ratio

APRs for physical IPV severity and highest education level reflect linear polynomial estimates

Severity levels of physical IPV are None, Moderate, and Severe

Education levels are None, Primary, and Post Primary

For the independent variables of interest, bolded measures indicate *p* ≤ 0.05

## Discussion

Gender-inequitable norms and structures disempower women and normalize behaviors that undermine their health, wellness, and ability to thrive. Our findings support the hypothesis that women who survive IPV are more likely to practice CCU as a strategy to regain reproductive autonomy. Among ever-sexually active women in rural Uganda, we observed that verbal and physical IPV were each positively associated with CCU, while sexual IPV’s relationship was marginally significant. These findings imply that the most disempowered women may use this tactic to avoid pregnancy in coercive and potentially hostile situations. These relationships may indicate that IPV compels women to initiate CCU, switch to more discreet methods, or employ secretive tactics to hide their contraceptive use [30, 31]. Since the prevalence of both IPV and CCU is unlikely to change without systemic population-level shifts in belief systems, women living in gender-inequitable environments will continue to require additional support and discretion to fulfill their fertility desires [32].

Polyvictimization was common among IPV survivors in this study. In the last 12 months, one-third of survivors experienced both verbal and physical IPV and another third endured all three forms. Individually, physical and sexual IPV often overlapped with other forms of violence. Another Ugandan study also found almost all IPV survivors endured physical and sexual polyvictimization. In that study, labor force participation was positively associated with polyvictimization, whereas wealth and educational attainment were negatively associated [33]. Our findings corroborate this pattern, highlighting the high levels of polyvictimization among women facing IPV. Evidence from South Africa and Nigeria also suggests that surviving multiple forms of violence can have detrimental effects on mental health, which in turn undermines interpersonal dynamics, relationship power, and self-efficacy [34, 35]. However, due to the cross-sectional nature of these studies, as well as our own analysis, it is impossible to ascertain if polyvictimization arose from co-occurring events or increasing IPV severity over time. To enhance the effectiveness of violence-reduction interventions, future studies should explore temporal pathways and interactions between different forms of violence to enhance intervention strategies. For example, verbal IPV had a high level of overlap with physical/sexual IPV in our sample and may serve as a precursor to more severe forms of IPV, making its experience an important marker for intervention and/or service referral. Moreover, since strangers, friends, and/or family members can also perpetrate violence, non-partner violence also warrants investigation to understand its effect on women’s health outcomes and trajectories.

Physical IPV had the strongest association with CCU, increasing the likelihood of reporting it by 39%. This result aligns with our prior exploratory study, which also found a strong association between physical IPV and CCU [13]. These findings are further corroborated by studies from other contexts. For example, physical IPV has consistently exhibited a strong positive association with CCU among married adolescent girls in Niger [17]. A mixed-methods study with Kenyan IPV survivors found a positive association between pregnancy coercion (including physical IPV) and CCU. Related qualitative insights indicated that women practiced CCU to avoid reproductive coercion but disclosing their CCU could lead to violence, including threats to forcibly remove contraceptive implants [30, 31]. Our results may reflect this dynamic or suggest that women switch to more discreet contraceptive methods—like injectables, used by 76% of the sample—to avoid further conflict after disclosing contraceptive use. Studies have also found that men perpetrate IPV when women attempt to initiate contraceptive discussions, leading some women to discontinue contraceptive use altogether or covertly practice CCU [31].

Interestingly, sexual IPV had the weakest association measure, increasing the likelihood of CCU by 22% and exhibiting only a marginally significant association. This finding is somewhat unexpected, as sexual IPV is the only form of violence that can directly result in unplanned or unwanted pregnancies, the very outcome that CCU seeks to prevent [36]. It is possible that the relationship was attenuated because survivors who became pregnant were excluded from our sample, especially considering Uganda’s strong abortion restrictions [37]. Hegemonic masculinity may have also led to underreporting, as some women may view sexual IPV as a typical aspect of marital relationships [11]. Sexual IPV’s relationship may have only achieved marginal significance due to unmeasured confounding, for we could not control for women’s attitudes regarding IPV and their potential acceptance of it. Given these complexities, qualitative inquiries are needed to better understand women’s perceptions of martial obligations, sexual violence, reproductive autonomy, and the overlap between these three topic areas, particularly in contexts where women have no/low power within relationships and contraceptive decision-making. Longitudinal interviews would help understand how women contextualize these phenomena over time, and supporting qualitative insights with quantitative data would better help explicate the social, cultural, relational, and intrapersonal drivers of these relationships. Other studies highlight that the relationship between sexual IPV and CCU remains empirically unclear. While the aforementioned Niger study found a strong positive association between physical IPV and CCU, it reported a definitively null relationship (*p* = 0.54) between sexual IPV and CCU [17] One Kenyan study found that married or partnered women who survived physical and/or sexual IPV had higher odds of CCU, but this comparison should be interpreted cautiously due to the aggregate IPV measure [38]. Since other studies measure or create samples using a broad IPV definition (e.g., experienced any form of IPV), it is difficult to compare them to our findings, suggesting that our study may provide some of the first insights into the specific relationships between sexual IPV and CCU in eastern Africa [30, 31]. Future studies should separately examine the influence of different IPV types to increase specificity and applicability for violence-reduction efforts.

Verbal IPV had the second-highest association with CCU, increasing the likelihood by 30%. Since our measure focused on verbal abuse and shouting, this form of IPV may reflect broader relationship dynamics, such as partners’ displeasure with contraception discussions, CCU, or perceived infertility [30, 31]. It may also serve as a proxy for other unreported IPV types, given the stigma and social desirability bias that can lead to underreporting of more severe IPV forms. Verbal IPV’s overlap with sexual IPV in polyvictimized survivors partially supports this hypothesis. The relationship between verbal IPV and CCU has been mixed in other studies. A multi-country study spanning Kenya, Cote d’Ivoire, and Burkina Faso found emotional IPV and CCU were only associated in Burkina Faso [38]. Although not a direct comparison, evidence from the United States elucidates that more women experience contraceptive interference when their partner is verbally abusive, signifying the potential need and justification for CCU [39]. Otherwise, verbal IPV is an underreported, understudied phenomenon due to societal normalization and/or increased focus on physical/sexual violence, reinforcing that further examination of this unreported phenomenon is warranted to guide intervention strategies [40].

The observed relationships may also reflect partners perpetrating IPV because they found out about CCU, either via discovery or purposeful disclosure. In-depth interviews with Ethiopian IPV survivors highlight that since IPV was expected when disclosing CCU, they devised violence-reduction strategies, like disclosing in the presence of other family members [30]. Qualitative data with women, men, providers, implementing partners, and general community members in Benin, Kenya, Malawi, Nigeria, and Uganda also support that CCU disclosure is often accompanied by IPV [6, 14]. Men’s justification for perpetrating IPV is largely rooted in gender-inequitable norms and power structures. While some believed CCU rightfully helped women autonomously enforce their fertility desires amid pervasive disempowerment, other men reacted to CCU with violence because it indicated infidelity, subverted their power, and inhibited their fertility desires [6, 14, 30].

Reducing IPV and CCU requires confronting and changing population-level acceptance of patriarchal norms [41]. These norms perpetuate economically and socially marginalizing practices, behaviors, and laws that disenfranchise women from societal systems and labor markets, undermining their financial stability, social and relational power, and agency [42]. With limited opportunities for social and economic independence, many women endure persistent IPV because of the support they receive from their partners, as the alternative may be destitution or survival sex work, which increases risk of HIV acquisition, unintended/unwanted pregnancy, and other adverse outcomes [43–45]. Although men are the primary instigators of IPV, some women also endorse gender-inequitable norms/practices, such as justifying women’s IPV experiences as acceptable. Thus, interventions must take a holistic approach, addressing both male and female endorsement of gender-inequitable practices [10]. Multi-level and multi-component interventions have shown some success in challenging community-level acceptance of IPV, but these normative shifts do not always translate into behavioral change. This implies that additional components are needed to catalyze normative shifts into meaningful behavior change [46, 47]. To optimize the development, delivery, and impact of interventions, there must be a greater understanding of the social and structural determinants of health and behavior across different ecological levels.

Healthcare systems should complement population-level interventions with improvements in clinical practices to enhance women’s reproductive autonomy. Women have reported that men can sabotage CCU by changing or destroying patient cards/records, which track clinic appointments [30, 31]. To prevent this, women recommend storing incriminating patient cards/materials at clinics. They also stress the importance of privacy, urging clinic workers to avoid inadvertently disclosing their CCU to unsupportive relatives or community members [22] Relatedly, some husbands and partners accompany their wives and partners to clinic visits to ensure they either decline contraception or discontinue CCU, a practice that should not occur without the woman’s consent. Healthcare providers must maintain confidentiality to avoid endangering women [30, 31]. In Ghana, providers have supported CCU by ensuring contraceptive methods are consistently and adequately stocked; establishing discreet ways for women to access clinics; offering flexible operation hours, including early mornings or late nights; and providing home visits, when requested [48]. Providers’ accommodations and discretion are integral to empowering women to safely and discreetly practice CCU, thereby regaining control over their reproductive health.

### Limitations

Despite its novel insights, this analysis has limitations. IPV may have been underreported due to social desirability bias, stigma, or respondents wanting to protect their partners. Causality cannot be inferred from this cross-sectional analysis. Additionally, CCU may have been underestimated by not including other discreet contraceptive methods (e.g., tubal ligation) in the survey’s skip pattern. Alternatively, CCU may be overreported since some of the methods included in the skip pattern have varying levels of discreetness and covert use was defined using participants’ responses, exclusively.

## Conclusion

In Uganda, women’s reproductive autonomy is often undermined by gender-inequitable norms, leading them to practice CCU to prevent pregnancy and fulfill their fertility desires. Because CCU is a marker of disempowerment, IPV likely influences CCU behaviors. Our findings showed that verbal and physical IPV were each positively associated with CCU, and sexual IPV was marginally associated with CCU. This evidence suggests that this tactic is utilized by the most disenfranchised women, emphasizing the need for additional understanding of the ecological factors, drivers, and norms that engender and reinforce women’s disempowerment within relationships and reproductive decision-making processes. Achieving Sustainable Development Goal 5 by 2030 requires swiftly leveraging actionable knowledge into robust, multi-level norm- and behavior-changing interventions, alongside infrastructural and operational improvements in health systems.

## Supplementary Information

Supplementary Material 1.

Supplementary Material 2.

## Data Availability

De-identified Rakai Community Cohort Study data can be provided to interested parties with a completed Rakai Health Sciences Program data request form and signed Data Transfer Agreement.
